# Effect of Organic Amendments in Soil on Physiological and Biochemical Attributes of *Vachellia nilotica* and *Dalbergia sissoo* under Saline Stress

**DOI:** 10.3390/plants11020228

**Published:** 2022-01-17

**Authors:** Muhammad Talha Bin Yousaf, Muhammad Farrakh Nawaz, Ghulam Yasin, Irfan Ahmad, Sadaf Gul, Muhammad Ijaz, Muhammad Zia-ur-Rehman, Xuebin Qi, Shafeeq Ur Rahman

**Affiliations:** 1 Department of Forestry and Range Management, University of Agriculture, Faisalabad 38000, Pakistan; talha.gorsi7@gmail.com (M.T.B.Y.); kf_uaf@yahoo.com (M.F.N.); irfan_uaf@yahoo.com (I.A.); 2 Department of Forestry, Range and Wildlife Management, The Islamia University Bahawalpur, Bahawalpur 63100, Pakistan; yasin_2486@yahoo.com; 3 Department of Forestry and Range Management, Bahauddin Zakariya University, Multan 60800, Pakistan; 4 Department of Botany, University of Karachi, Karachi 75270, Pakistan; sadafgpk@yahoo.com; 5 Bahadur Sub Campus Layyah, College of Agriculture, Bahauddin Zakariya University, Multan 60800, Pakistan; muhammad.ijaz@bzu.edu.pk; 6 Institute of Soil and Environmental Sciences, University of Agriculture, Faisalabad 38000, Pakistan; ziasindhu1399@gmail.com; 7 Farmland Irrigation Research Institute, Chinese Academy of Agricultural Sciences, Xinxiang 453000, China; 8 School of Environment and Civil Engineering, Dongguan University of Technology, Dongguan 523000, China

**Keywords:** soil degradation, afforestation, salinity, nursery raising, agroforestry

## Abstract

*Vachellia nilotica* (L.) P.J.H. Hurther & Mabb. and *Dalbergia sissoo* Roxb. are two of the most important multipurpose agroforestry tree species of the Indian sub-continent, but their growth in saline soils is greatly reduced. Recently, organic amendments have showed the potential to increase plant growth in salt-affected soils; however, the influence of using these amendments for growing the above-mentioned tree species under saline conditions is not yet quantified. Therefore, an experiment was devised to analyze the interactive effects of organic amendments in saline soils on the growth of *V. nilotica* and *D. sissoo*. Under controlled conditions, a pot experiment was conducted in sandy loam saline soils (EC = 20.5 dSm^−1^). Organic amendments from four diverse sources: farmyard manure (FYM), poultry manure (PM), slurry (SL), and farmyard manure biochar (FYMB) were employed in this study. At the harvesting time, data regarding morphological, physiological, ionic, and biochemical parameters were obtained. The current study results indicated that both tree species reacted differently, but positively, to diverse applied amendments. The maximum increment in total above-ground biomass, total below-ground biomass, and shoot length for *V. nilotica* (163.8%, 116.3%, and 68.2%, respectively) was observed in FYM amended soils, while the maximum increment for *D. sissoo* (128%, 86%, and 107%, respectively) was observed in FYMB amended soils, as compared to control. Minimum plant growth of both species was observed in untreated soils (saline soils). Likewise, the maximum potassium ion and minimum sodium ion concentrations were present in the root and shoots of plants (both species) treated with FYMB. The use of organic amendments resulted in decreased concentrations of malondialdehyde and hydrogen peroxide, and increased concentrations of antioxidant enzymes such as SOD, POD, and CAT. Moreover, higher photosynthetic rates and stomatal conductance were observed in the plants grown in amended soils. The findings of this study can be used to include the above-mentioned high-value tree species for future afforestation programs under saline conditions.

## 1. Introduction

Salinity is a global issue, and the area affected by salinity is increasing persistently [1,2]. In a few countries, salinity and sodicity affect more than 50% of the cultivated land [3]. The productive area of the world is decreasing due to salinity and other abiotic stresses while the world population is increasing, raising the issue of food security [2,4]. There is therefore a dire need to find ways to make these lands productive [5,6]. Many approaches can reclaim saline soils such as engineering, chemical, and biological techniques [2,7]. Using green plants is an efficient, sustainable, and cost-effective practice to improve salt-affected soils’ quality [8,9]. Moreover, among vegetation, trees are considered to have more potential due to their higher tolerance index, larger biomass production, and longer periods of growth [9,10,11,12,13,14,15,16]. However, only selected tree species can be grown on saline soils [17].

Under sub-tropical and semi-arid conditions, several tree species have been reported to survive under light to moderate salinity levels, such as *Vachellia nilotica*, *Vachellia auriculiformis*, *Albizia lebbek*, *Terminalia arjuna*, *Prosopis juliflora*, *Casuarina equisetifolia*, *Prosopis cineraria*, *Tamarix indica*, *Dalbergia sissoo*, *Eucalyptus camaldulensis*, *Emblica officinalis*, *Zizyphus jujube*, and *Syzygium cumini* [18,19]. Among them, *V. nilotica* and *D. sissoo* are farm-friendly tree species found in several parts of the world, particularly in India, Pakistan, Sri Lanka, Nepal, Australia, and Africa [20,21]. Both are highly valuable for timber, furniture, fodder, medicinal value [22], and other environmental services such as climate change mitigation, shade, beauty, soil fertility improvement, etc. [23,24]. Both tree species also play an important role in environmental conservation and ecosystem balance [25]. These species are reported to have the ability to tolerate saline conditions and ameliorate the soil’s physicochemical properties [26,27]. Trees have the ability to minimize salt deposition and salt accumulation in the upper layer of soil due to higher transpiration rate of trees and lower evaporation rate under the shade of trees, respectively [28,29]. The deep root system of trees helps in improving the physical structure of soil and allows the translocation of sodium ions in deeper soil horizons [30,31]. However, in these types of degraded soils, trees have to face various physiological problems and their growth is reduced [19,28].

Generally, saline soils have low organic matter content [32]. However, it is recently reported that several organic amendments made from agricultural wastes such as green manures, farmyard and poultry manure, agro-industrial by-products, food processing wastes, mulches, cover crops, and composts can be very helpful in improving soil health and plant growth under saline conditions [33,34,35,36]. Farmyard manure has been used to ameliorate degraded soils for several centuries and it has the potential to mitigate the negative impacts of salinity, enhance plant biomass, and increase yield [37]. Similarly, poultry manure and slurry have shown very positive results in enhancing vegetative cover on salt-degraded lands [38,39]. Biochar, a processed form of the organic amendment, has shown promising results and has resulted in increased crop yields and plant growth under saline conditions [36,40]. It is considered one of the most important discoveries in the history of mankind [40,41,42,43]. There are different types of biochar made from diverse sources under characteristic environmental conditions [9,16,41,44]. In another experiment, different types of biochar were applied to check their effect on the growth of three different agroforestry tree species. Among three different types of biochar, farmyard manure biochar showed the best results. It not only improved soil conditions, but also enhanced plant growth [9].

This experiment was devised to assess the impact of commonly available organic amendments (FYM, PM and SL) and processed organic amendment in the form of biochar (FYMB) on the growth of two important farm-friendly tree species: *V. nilotica* and *D. sissoo* under saline conditions. The result of using various organic amendments on the growth and physiological attributes of selected species were studied, and the combined ameliorative effect of the selected amendments and species on soil physicochemical properties was also studied.

## 2. Results and Discussion

### 2.1. Growth Attributes

**Figure 1:**
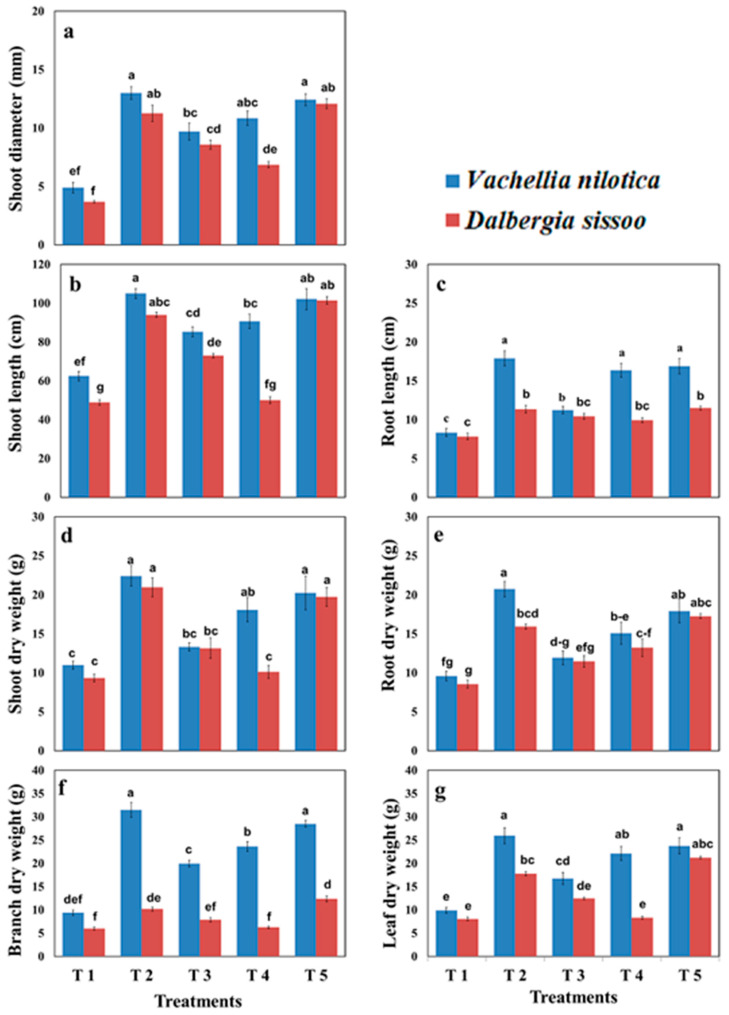
Effects of different organic amendments on (**a**) shoot diameter, (**b**) shoot length, (**c**) root length, (**d**) shoot dry weight, (**e**) root dry weight, (**f**) branch dry weight, and (**g**) leaf dry weight of *Vachellia nilotica* and *Dalbergia sissoo,* where T1 = control, T2 = farmyard manure, T3 = poultry manure, T4 = slurry, T5 = biochar. The lower case letters the indicate that values are significantly (*p* < 0.05) different from each other.

Figure 1 depicts the growth parameters of the selected two species with five different treatments. *V. nilotica* generally showed better results as compared to *D. sissoo* for all growth parameters. There was a 68%, 36%, 45%, and 63% increase in the shoot length of *V. nilotica* in T2, T3, T4, and T5, respectively, as compared to T1. There was a 92%, 79%, 2.2%, and 107% increase in the shoot length of *D. sissoo* in T2, T3, T4, and T5, respectively, as compared to T1. There was a 114%, 34%, 96%, and 102% increase in the root length of *V. nilotica* in T2, T3, T4, and T5, respectively, as compared to T1. There was a 45%, 33%, 26%, and 42% increase in the root length of *D. sissoo* in T2, T3, T4, and T5, respectively, compared to T1. There was a 163%, 65%, 110%, and 139% increase in the total above-ground biomass of *V. nilotica* in T2, T3, T4, and T5, respectively, as compared to T1. There was a 109%, 45%, 5%, and 128% increase in the total above-ground biomass of *D. sissoo* in T2, T3, T4, and T5, respectively, as compared to T1. There was a 116%, 24%, 57%, and 86% increase in the below-ground biomass of *V. nilotica* in T2, T3, T4, and T5, respectively, compared to T1. There was an 86%, 34%, 55%, and 102% increase in the below-ground biomass of *D. sissoo* in T2, T3, T4, and T5, respectively, as compared to T1. The general trend of *V. nilotica* with different treatments for all growth parameters was recorded as T2 > T5 > T4 > T3 > T1. The general trend of *D. sissoo* with different treatments was recorded as T5 > T2 > T3 > T4 > T1.

### 2.2. Physiological Attributes

**Figure 2:**
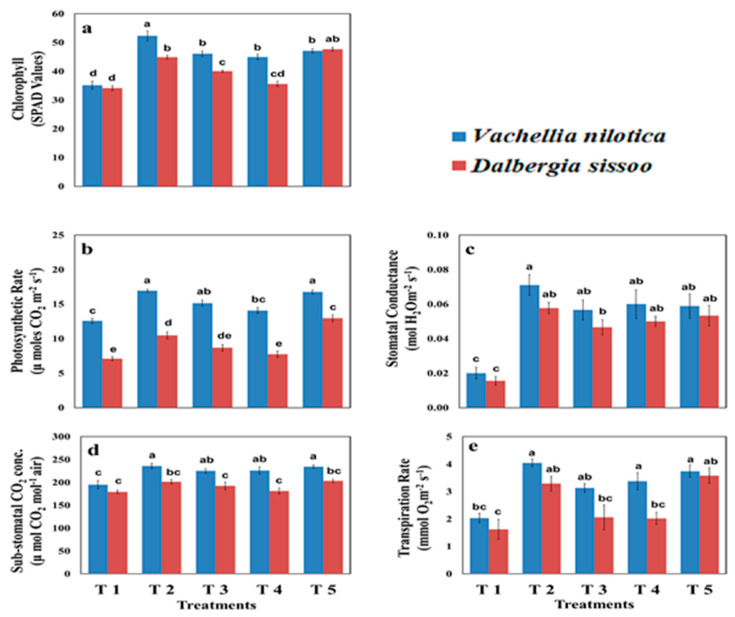
Effect of different organic amendments on (**a**) chlorophyll content, (**b**) photosynthetic rate, (**c**) stomatal conductance, (**d**) substomatal CO_2_ concentration, (**e**) transpiration rate of *Vachellia nilotica* and *Dalbergia sissoo*, where T1 = control, T2 = farmyard manure, T3 = poultry manure, T4 = slurry, T5 = biochar. The lower case letters indicate that values are significantly (*p* < 0.05) different from each other.

Figure 2 depicts the effect of different organic amendments on physiological characteristics of *V. nilotica* and *D. sissoo*. All treatments affected the chlorophyll concentrations of plants. FYM and FYMB showed maximum SPAD values for chlorophyll contents. Chlorophyll contents of *Vachellia nilotica* increased by 48%, 31%, 28%, and 34% in T2, T3, T4, and T5, respectively, as compared to control. Chlorophyll contents of *Dalbergia sissoo* increased by 31%, 17%, 10%, and 40% in FYM, PM, SL, and FYMB, respectively, as compared to control. The photosynthetic rate of both species was increased by using all types of organic amendments. The photosynthetic rate was increased by 34%, 20%, 12%, and 33% for *V. nilotica* against FYM, PM, SL, and FYMB, respectively. The photosynthetic rate was increased by 33%, 17%, 9%, and 36% for *D. sisso* in T2, T3, T4, and T5, respectively. The maximum stomatal conductance (0.07 mol m^−2^ s^−1^) and sub-stomatal CO_2_ (236.11 µmol m^−2^ s^−1^) were exhibited by the *V. nilotica* plants treated with farmyard manure. The minimum stomatal conductance (0.02 mol m^−2^ s^−1^) and sub-stomatal CO_2_ (179.44 µmol m^−2^ s^−1^) were shown by the *D. sissoo* plants with the control treatment. The general trend of *V. nilotica* with different treatments for all physiological parameters was recorded as T2 > T5 > T4 > T3 > T1. The general trend of *D. sissoo* with different treatments was recorded as T5 > T2 > T3 > T4 > T1.

### 2.3. Sodium and Potassium Contents in Plants

The sodium (Na) and potassium (K) concentrations were determined for shoots and roots for both species in all treatments. The Na contents in shoots of *V. nilotica* were decreased by 45%, 31%, 33%, and 79% in T2, T3, T4, and T5, respectively, compared to control. The concentration of Na in shoots of *D. sissoo* was decreased by 53%, 33%, 35%, and 83% in T2, T3, T4, and T5, respectively, as compared to control.

**Figure 3:**
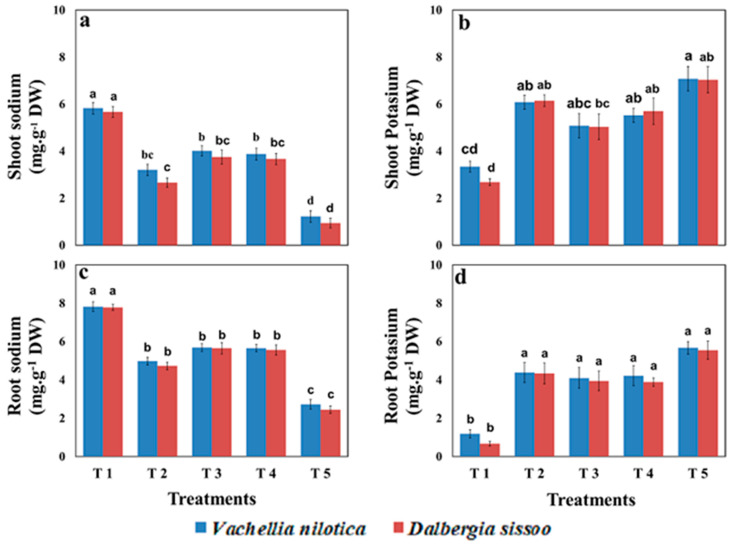
Effect of different organic amendments on (**a**) shoot sodium, (**b**) shoot potassium, (**c**) root sodium, and (**d**) root potassium of *Vachellia nilotica* and *Dalbergia sissoo*, where T1 = control, T2 = farmyard manure, T3 = poultry manure, T4 = slurry, T5 = biochar. The difference between lower case letters is the indication that values are significantly (*p* < 0.05) different from each other.

The K contents (mg g^−1^ DW) in shoots of V. nilotica were increased by 81%, 51%, 65%, and 111% in T2, T3, T4, and T5, respectively, compared to control. The K contents (mg g^−1^ DW) in shoots of *D. sissoo* were increased by 129%, 87%, 112%, and 62% in T2, T3, T4, and T5, respectively, compared to control. Minimum sodium and maximum potassium concentrations in roots of *D. sissoo* and *V. nilotica* were recorded in the T5 treatment. The results of sodium and potassium concentration are illustrated in Figure 3.

### 2.4. Biochemical Parameters

**Figure 4:**
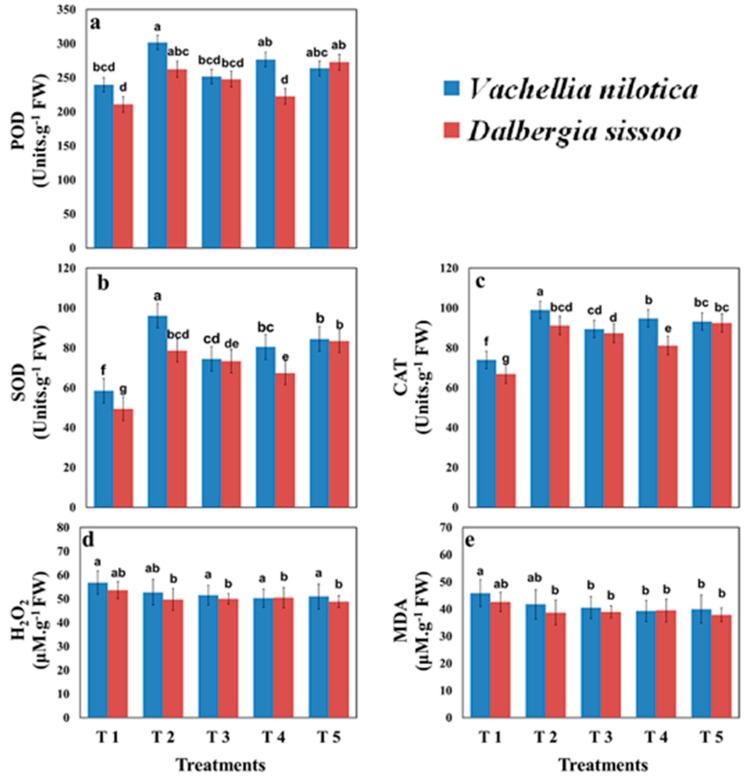
Effect of different organic amendments on (**a**) peroxidase (POD), (**b**) superoxide dismutase (SOD), (**c**) catalase (CAT) activities, concentrations of (**d**) hydrogen peroxide (H_2_O_2_) in plants’ leaves, and (**e**) malondialdehyde (MDA). T1 = control, T2 = farmyard manure, T3 = poultry manure, T4 = slurry, T5 = biochar. The difference between lower case letters is the indication that values are significantly (*p* < 0.05) different from each other.

Figure 4 depicts the results of the effect of different organic amendments and selected tree species on selected enzymatic activities. Results indicated that all types of organic amendments enhanced antioxidant enzymatic activities as compared to control. Higher concentrations of H_2_O_2_ and MDA (μM g^−1^ FW) were found in the plants of control treatment, whereas organic amendments reduced the concentration of H_2_O_2_ and MDA, but results were not statistically significant for different types of organic amendments. The minimum concentration of antioxidant enzymatic activities was present in the *D. sissoo* plants. The maximum increments in SOD (68.76%) and CAT (38.20%) activities were found in the *D. sissoo plants* with the T5 treatment. POD activities were not affected significantly by using different organic amendments.

### 2.5. Post-Harvest Soil Characteristics

The current study results demonstrate that post-harvest characteristics of soils are significantly affected by using different types of organic amendments. The minimum value of pH was observed in the plants of control treatment; although pH was significantly reduced as compared to initial soil characteristics, there was no significant difference among different treatments for the value of pH after harvesting of plants.

Maximum electrical conductivity was found in the plants of the control treatment. All types of applied organic amendments showed very promising results in reducing the values of EC and improving the physicochemical characteristics of the soil. All types of organic amendments were found to be helpful in reducing the values of the total soluble salts (TSS) and the sodium adsorption ratio (SAR). The use of organic amendments also improved the organic matter and saturation percentages. It was also observed that the concentration of potassium and calcium ions was increased and the concentration of sodium ions was decreased by applying different types of organic matter as a soil amendment. Among different types of applied organic amendments, biochar showed the most promising results in reducing the values of EC, TSS, SAR, and sodium ions. Furthermore, biochar was also the best amendment to improve the organic matter contents of soil, saturation percentage, and concentration of potassium ions. Data regarding different post-harvest soil characteristics are given in Table 1. We can say that all types of applied organic amendments were beneficial in improving the physicochemical characteristics of soil, but biochar showed the best results.

Among trees species, *Vachellia nilotica* showed the best results as compared to *Dalbergia sissoo* in terms of improved physicochemical characteristics, as it can be seen from the table that the EC reduced from 20.5 dS/m–12.41 dS/m in the soil where *D. sissoo* was grown without any amendment, whereas the EC of soil decreased from 20.5 dS/m to 8.71 dS/m of untreated *V. nilotica* soil. Similar results were obtained for all other parameters. These results show that the phytoremediation potential of *V. nilotica* is greater than *Dalbergia sisoo* for ameliorating salt degraded lands and biochar is the best amendment to improve the physicochemical properties of soil.

The results of this study represent the salinity tolerance potential of two important agroforestry species (*Vachellia nilotica* and *Dalbergia sissoo*) and, at the same time, the effects of three commonly found organic wastes (farmyard manure, poultry manure, and slurry) and one processed organic amendment, in the form of biochar, on the growth and physiology of these species along with the physicochemical properties of soil. Results indicated that salinity negatively affected the plant’s growth. Almost all morphological and growth parameters such as shoot length, root length, dry weight of root, shoots, leaves, and branches were less in the control treatment plants (under salinity). The reduction in growth is due to a higher concentration of salts in saline soils. The osmotic potential of salt-affected soils is low and when plants are grown in this soil, they must keep their internal osmotic potential low to avoid exosmosis. This, in turn, leads to oxidative stress and dehydration [45,46,47,48]. Salinity stress also causes nutrient imbalance, and nutrients such as N, Ca, K, and toxic ions replace P, Fe, Zn, and as a result, their uptake to shoot is decreased [49,50,51].

Different species responded to salinity in different ways, as *Vachellia nilotica* showed better growth as compared to *Dalbergia sissoo* and was found to be more effective in the remediation of saline soils. The response of plants to salinity is species and genotype-dependent [52]. This may be due to difference in the root behavior and hardiness of these species towards such an adverse soil environment. The differences in the salinity tolerance in these two species may be due to different rates of salt transport to the shoots, which adversely affects leaf growth, reducing the photosynthetic efficiency of the plants [53]. In another experiment, seedlings of *Vachellia nilotica* and *Acacia ampliceps* were grown under different salinity levels of up to 400 mM NaCl concentration and all growth parameters were reduced at the time of harvesting [20].

Although all the amendments showed better results for both species than control, both species’ responses to different amendments were different. Farmyard manure showed the most promising results for the growth of *V. nilotica*, whereas FYMB was the best amendment for the growth of *D. sissoo*. Likewise, slurry showed better results for *V. nilotica* as compared to poultry manure, but results were the opposite for *D. sissoo*, where poultry manure showed better results than slurry. Therefore, we can say that response of plants to different organic amendments is species-specific. It was reported that the response of six different woody seedlings was species-specific under organic fertilization [54]. Likewise, five different ornamental plant species responded differently to applying the same organic matter [55]. In another study, a meta-analysis was performed and it was reported that the first-year crop yield of different agronomic crops is different for different commonly used organic amendments [56]. Plant growth of *Corymbia maculata* and *Eucalyptus torquata* was not the same for applied organic amendments. *Eucalyptus torquata* showed better results than control after using organic amendments, whereas *Corymbia maculate* did not show any significant results after applying the same amendment [57]. Among different types of biochar, sugarcane bagasse biochar showed the better results for the growth of *D. sissoo*, whereas woodchips biochar was more effective for the growth of *V. nilotica* [9].

The current study results revealed that minimum Na and maximum K concentrations were found in the plant parts treated with biochar. Biochar improved potato crop yield by decreasing Na contents and increasing K contents [58]. Biochar is reported to significantly enhance the concentration of potassium in leaf sap of maize [59]. Biochar showed the ability to increase the amount of available potassium and reduce the bioavailability of sodium ions [60]. Biochar application increased the available K and resultantly enhanced the biomass of lentils [61]. Biochar has the ability to reduce toxic elements by the process of adsorption [62]. Salt-affected soil amended with farmyard and poultry manures for rice showed enhanced growth and yield, increased amounts of potassium and K/Na, and increased salt-tolerance [63].

The use of organic amendments increased antioxidant enzymatic activities (SOD, POD, and CAT). This shows the enhanced tolerance of plants to salinity stress [64]. The antioxidant defense system of spinach leaves was also increased by using biochar and other amendments [65]. Moreover, different organic and inorganic amendments significantly increased antioxidant enzymatic activities in Zea mays against cadmium contamination [66]. In addition, the use of organic amendments, including biochar, improved mung bean antioxidant defense systems [67].

All amendments showed better results for chlorophyll contents and other gas exchange parameters for both species compared to control. It was reported that biochar increased the stomatal conductance and photosynthetic rate of okra plants [68], fava beans [69], and maize plants [70]. Photosynthetic activity and other physiological parameters of plants are also reported to be enhanced by the use of organic amendments [71].

All organic amendments generally improved soil physicochemical characteristics of saline soils, with biochar showing the best results in this study. Suppose that organic amendments are used to treat salt-affected soils. In that case, they have several benefits to improve the quality of soil, such as increased water holding capacity [72], enhanced soil fertility [73], increased organic carbon [74,75], improved cation exchange capacity (CEC), decreased electrical conductivity [9,76,77], structural stability, [78,79], reduction in soil compaction [80], and enhancement of different enzymatic activities such as urease, alkaline phosphatase, and catalase activities [81,82].

Biochar application reduced the uptake of sodium ions (Na^+1^), enhanced uptake of potassium ions (K^+^) [83,84,85], influenced processes of Na^+1^ leaching, and decreased the sodium adsorption ratio (SAR) and electrical conductivity (EC) [84,86,87].

When coastal saline soils are treated with biochar, sodium can be substituted by beneficial ions such as potassium, magnesium, and calcium on the soil colloids [88]. Soil chemical properties such as pH and CEC were significantly affected by the interactive effect of biochar and farmyard manure, and available potassium was also increased [19,89].

## 3. Materials and Methods

An experimental area of the Department of Forestry and Range Management (FRM), University of Agriculture Faisalabad (UAF) (31°25′57″ N, 73°04′21″ E) was selected for this experiment. Two-month-old seedlings of *Vachellia nilotica* and *Dalbergia sissoo* were planted in earthen pots. This experiment was conducted for a period of six months. Climatic conditions during the experiment are given in Table 2.

Soil samples from different localities of Faisalabad district were collected and analyzed, and soil with low productivity and high electrical conductivity value was chosen for this study. The procedure of selecting the fields for soil collection and preparation of soil for experiments was discussed earlier in [19]. Sandy loam soil with 60% sand, 25% silt, and 15% clay was used in this experiment. Different soil physicochemical characteristics were determined according to the procedures described by the US Salinity Laboratory Staff 1954 [90]. The hydrometer method described by [91] was followed for the determination of soil texture. The pH was measured using a Jenway pH meter (Model-671P) following calibration with two buffer solutions of pH 4.00 and 9.00. The EC was measured using a Jenway conductivity meter (Model-4070) following calibration with a 0.01 N KCl solution. The EC was converted into total soluble salts using the graph on page 12 of the USDA Handbook No. 60 [90].

Soluble Ca and Mg were determined by titrating the saturation extract with 0.01 N EDTA (disodium) solution to a blue endpoint using Erichrome Black T indicator in the presence of NH_4_OH + NH_4_Cl buffer solution. Soluble Na and K were determined using a Jenway PFP-7 flame photometer with Na or K filters in place. The instrument was calibrated with a series of Na or K (0–20 ppm) standards. Standards graphs were separately plotted for Na and K containing standards and flame photometer readings. Carbonate content was determined by titrating a saturation extract with 0.01 N H_2_SO_4_ to a colorless endpoint using Phenolphthalein as an indicator. Bicarbonate content was determined by titrating an aliquot obtained after CO_3_^2−^ determination with 0.01 N H_2_SO_4_ to a pinkish yellow endpoint using methyl orange as an indicator. Soluble chloride content was determined by titrating the aliquot after HCO_3_^−^ determination with 0.01 N AgNO_3_ solution to a brick red endpoint using potassium chromate (K_2_CrO_4_) as an indicator. Soil OM was determined following the Walkly–Black method described in [92]. The characteristics of the soil used in this experiment are given in Table 3.

Four types of commonly available organic amendments—farmyard manure (FYM), poultry manure (PM), slurry (SL), and farmyard manure biochar (FYMB)—were applied in this experiment. Farmyard manure (FYM) is a fertilizer composed of waste products, typically dung and urine, produced by farm animals, most commonly cows and buffaloes. Poultry manure (PM), also known as chicken manure, mainly comprised of feces of chicken, is also an excellent soil amendment. Slurry (SL) is obtained as a byproduct of biogas. A biogas digester/reactor converts dung into biogas and bioslurry is produced.

These amendments were collected from the farm area of the agronomy department of the UAF. Dung cakes were used as feedstock material to prepare biochar. Biochar was prepared at 450 °C for the duration of 3 h under slow pyrolysis conditions.

These organic amendments were thoroughly mixed with the soil of the selected field at the rate of 6% (*w*/*w*). Characteristics of the amendments used in this experiment are given in Table 3.

Uniform-sized, healthy, and disease-free seedlings of the *Vachellia nilotica* (SP 1) and *Dalbergia sissoo* (SP 2) were collected from the nursery of the Department of FRM, UAF. They were planted in pots containing soil mixed with organic amendments. The internal diameter and height of the pots were 10 inches, and each pot contained 10 kg of soil. Tap water with pH (7.29), EC (0.669 dS/m), and RSC (1.8) was used to irrigate the plants from the nursery of the Forestry Department, UAF. Plants were irrigated with almost a half-liter of water on alternate days for each pot during the first two months. After that, from May to August, plants were irrigated with a half-liter of water on a daily basis.

The total number of treatments for this experiment was five: control (T1), FYM (T2), PM (T3), SL (T4), and FYMB (T5). There were three replications of each treatment, and for each replication, three plants were selected. Thus, nine plants were selected for each treatment for both species.

Vernier calipers and measuring tape were used to measure shoot diameter and shoot length of plants before harvesting. Both plant species’ growth parameters and morphological characteristics were determined according to the earlier patterns [16,19,93]. An LCA-4 ADC portable infrared gas analyzer (Analytical Development Company, Hoddesdon, England) was used to measure the gas exchange parameters. Each plant’s second upper fully expanded leaf was selected to measure photosynthetic parameters such as photosynthetic rate, stomatal conductance, sub-stomatal conductance, and transpiration rate measured according to the procedures described earlier in [9,94]. To ensure unchanged photon flux density and temperature, readings were made at a similar time each day (between 10:00 a.m. and 12:00 p.m.) by clamping the central part of the leaf in the chamber of the instrument. In terms of SPAD values, chlorophyll measurement was also performed on the third upper leaf using a chlorophyll meter (SPAD-502, Konika Minolta Sensing Inc., Tokyo, Japan).

To estimate hydrogen peroxide content in leaves, 50 mg of leaf sample was taken and standardized with 3 mL of phosphate buffer solution. This sample was then mixed with 1 mL of titanium sulphate in 20% (*v*/*v*) H_2_SO_4_. Next, this mixture was subjected to a centrifugation process at 6000× *g* resolution for a period of 15 min. After that, the absorption of the supernatant was calculated using a spectrophotometer at the wavelength of 410 nm. To calculate malondialdehyde contents of leaves, a 0.2 g leaf sample was homogenized in 5 mL of trichloroacetic acid (TCA). After that, this sample was passed through the process of centrifugation at 10,000× *g* of resolution for a period of 20 min. In the end, the absorption of supernatant was calculated at three different wavelengths, namely 450, 532 and 600, to estimate leaf MDA contents by using the following equation. The details of the determination of the biochemical parameters are given in [94,95].

C (μmol L^−1^) = 6.45(A532 − A600) − 0.56 A450

0.2 g leaf samples were taken and mixed with 0.05 M buffer solution of phosphate to determine the activities of SOD and POD. The mixture was passed through the process of centrifugation at 12,000× *g* resolution for a period of 20 min. The detailed procedure for determining the activities of SOD and POD is described earlier in [96,97], while CAT activity was determined according to the procedure described earlier in [98].

Roots, shoots, and leaves were separately oven-dried at 75 °C till the constant weight was obtained. Oven-dried plant material was ground and then digested using nitric acid and perchloric acid. According to the procedures explained earlier, sodium and potassium contents in different plant parts were determined after digestion, using a Jenway-PFP 7 flame photometer [9,99].

## 4. Statistical Analysis

Two factor factorial analysis under CRD was used to analyze the data with the help of the computer-based software Statistix 8.1 (Tallahassee, FL, USA). There were two factors i.e., species and treatments. Their interaction effect was determined using the ANOVA technique, and the significance of treatments was calculated using Tukey’s HSD Test.

## 5. Conclusions

Organic amendments showed a positive effect on the growth of *Vachellia nilotica* and *Dalbergia sissoo* under saline conditions. Organic amendments enhanced enzymatic activities, reduced oxidative stress, increased potassium concentration, and decreased sodium concentration, resulting in improved growth of both plant species. At the seedling stage, farmyard manure showed better results for the growth of *Vachellia nilotica*, whereas biochar was the most effective amendment for the growth of *Dalbergia sissoo*. A long-term field trial is required to determine field feasibility with cost analysis for planting these trees species under recommended amendments in saline soils. Moreover, the effects of different biochar and farmyard manure levels on the growth of *Dalbergia sissoo* and *V. nilotica* should also be tested. The findings of this study can be employed during afforestation and reforestation programs, mainly when performed with the prime objective of saline soil reclamation.

## Figures and Tables

**Table 1:** Effect of various organic amendments and agroforestry trees species on post-harvest soil characteristics.

Parameters	T1		T2		T3		T4		T5	
	SP 1	SP 2	SP 1	SP 2	SP 1	SP 2	SP 1	SP 2	SP 1	SP 2
pH	7.77a ± 0.04	7.77a ± 0.04	7.87a ± 0.04	7.88a ± 0.04	7.87a ± 0.04	7.89a ± 0.04	7.85a ± 0.04	7.87a ± 0.04	7.84a ± 0.04	7.84a ± 0.04
EC (dS/m)	8.71b ± 0.2	12.4a ± 0.2	3.94de ± 0.2	8.42b ± 0.2	6.83c ± 0.2	8.75b ± 0.2	4.13de ± 0.2	8.15b ± 0.2	3.57e ± 0.2	4.53e ± 0.2
TSS(mmol_c_/L)	87.10b ± 2.1	124.1a ± 2.1	39.3de ± 2.1	84.2b ± 2.1	68.3c ± 2.1	87.5b ± 2.1	41.3de ± 2.1	81.5b ± 2.1	35.7e ± 2.1	45.2e ± 2.1
HCO_3_^−^(mmol_c_/L)	4.44a ± 0.3	4.51a ± 0.3	3.2b ± 0.3	3.5ab ± 0.3	3.6b ± 0.3	3.7ab ± 0.3	3.2ab ± 0.3	3.8ab ± 0.3	3.2b ± 0.3	3.5ab ± 0.3
Cl^−^ (mmol_c_/L)	51.0b ± 1.7	60.0a ± 1.7	19.3fg ± 1.7	28.6cde ± 1.7	31.3cd ± 1.7	34.6cd ± 1.7	22.6efg ± 1.7	27.3de ± 1.7	17.3g ± 1.7	25.4def ± 1.7
Ca^2+^ + Mg^2+^ (mmol_c_/L)	3.7 c ± 0.7	3.83bc ± 0.7	7.5a ± 0.7	7.5a ± 0.7	7.1a ± 0.7	6.6a ± 0.7	6.4ab ± 0.7	6.6ab ± 0.7	5.0abc ± 0.7	5.2abc ± 0.7
Na^+^ (mmol_c_/L)	51.0b ± 1.2	62.33a ± 1.2	22.3fg ± 1.2	35.0d ± 1.2	28.0e ± 1.2	37.3cd ± 1.2	25.0e ± 1.2	40.3c ± 1.2	18.5h ± 1.2	19.9gh ± 1.2
SAR	37.4b ± 1.3	45.5a ± 1.3	11.7e ± 1.3	18.0cd ± 1.3	14.9de ± 1.3	20.6c ± 1.3	13.9de ± 1.3	22.1c ± 1.3	11.6e ± 1.3	12.3e ± 1.3
K^+^(mmol_c_/L)	6.5c ± 0.4	6.0c ± 0.4	11.3a ± 0.4	11.4a ± 0.4	10.3ab ± 0.4	10.4ab ± 0.4	9.5b ± 0.4	9.4 b ± 0.4	11.6a ± 0.4	12.0a ± 0.4
OM (%)	0.51e ± 0.1	0.50e ± 0.1	0.69bc ± 0.1	0.66cd ± 0.1	0.64cd ± 0.1	0.63d ± 0.1	0.62d ± 0.1	0.63d ± 0.1	0.77a ± 0.1	0.74ab ± 0.1
Saturation (%)	34.5d ± 0.47	34.5d ± 0.5	39.6b ± 0.5	38.8b ± 0.5	37.6c ± 0.5	37.6c ± 0.5	38.5bc ± 0.5	38.5bc ± 0.5	45.4a ± 0.5	45.3a ± 0.5

Mean value (*n* = 3); ± standard error; significant difference based on *p* < 0.05; the lower case letters indicate that values are significantly (*p* < 0.05) different from each other. T1, control; T2, farmyard manure; T3, poultry manure; T4, slurry; T5, Biochar. SP1 = *Vachellia nilotica*, SP2 = *Dalbergia sissoo*.

**Table 2:** Climatic conditions data during experimental months.

Month	Average Max. Temp. (°C)	Average Min. Temp. (°C)	Precipitation (mm)	Sunshine Duration (Hours)	ET₀ (mm)
March	27.3	14.2	16.2	07.2	02.7
April	37.7	20.9	28.3	09.2	05.2
May	41.1	26.0	10.1	10.4	05.7
June	39.8	27.3	41.6	09.38	05.3
July	38.5	28.9	117.2	07.0	04.0
August	38.1	28.6	66	07.9	03.8

**Table 3:** Analysis of selected parameters for soil and other organic amendments used in the experiment.

Parameters	Soil	FYM	PM	SL	FYMB
pH	8.5 ± 0.3	5.8 ± 0.2	6.3 ± 0.3	6.0 ± 0.3	7.0 ± 0.2
EC (dS/m)	20.5 ± 9	6.55 ± 2.5	7.31 ± 3.2	6.82 ± 3.8	2.08 ± 0.34
TSS (mg/kg)	205 ± 20	65 ± 5.8	73.1 ± 10.2	68.2 ± 8.4	20.8 ± 4.2
CO_3_^2−^ (mg/kg)	10 ± 2	(-)	(-)	(-)	(-)
HCO_3−_ (mg/kg)	30 ± 4	10 ± 3.2	6 ± 3.8	12 ± 4.5	8 ± 2.5
Cl^−^ (mg.kg^−1^)	140 ± 19	8.0 ± 2.2	5 ± 3.2	5 ± 3.2	4 ± 2.1
Ca^2+^ + Mg^2+^ (mg/kg)	12 ± 4.5	7.3 ± 4.2	6.3 ± 3.1	6.9 ± 2.5	13.2 ± 2.2
Na^+^ (mg/kg)	160 ± 15.2	11.6 ± 1.8	15.2 ± 3.4	13.4 ± 2.5	3.4 ± 0.8
K^+^ (mg/kg)	47 ± 7	9.2 ± 2.7	8.8 ± 2.5	8.9 ± 2.9	7.2 ± 2.2
OM (%)	0.64 ± 0.03	50.8 ± 2.4	67.2 ± 5.5	65.4 ± 4.3	95.4 ± 3.6

## Data Availability

Data available on request from the corresponding author.
